# Supplementary material for: Honey-collecting in prehistoric West Africa from 3500 years ago
Source: Nat Commun. 2021 Apr 14;12:2227. doi: 10.1038/s41467-021-22425-4 (PMC8047003; doi:10.1038/s41467-021-22425-4)
Supplement: Supplementary file 2 — Reporting Summary [file 41467_2021_22425_MOESM2_ESM.pdf]

Corresponding author(s): Dr J Dunne

Last updated by author(s): 18/02/2021

Reporting Summary

Nature Research wishes to improve the reproducibility of the work that we publish. This form provides structure for consistency and transparency in reporting. For further information on Nature Research policies, see our Editorial Policies and the Editorial Policy Checklist.

Statistics

For all statistical analyses, confirm that the following items are present in the figure legend, table legend, main text, or Methods section.

n/a	Confirmed	
☐	☒	The exact sample size (n) for each experimental group/condition, given as a discrete number and unit of measurement
☐	☒	A statement on whether measurements were taken from distinct samples or whether the same sample was measured repeatedly
☒	☐	The statistical test(s) used AND whether they are one- or two-sided<br>*Only common tests should be described solely by name; describe more complex techniques in the Methods section.*
☒	☐	A description of all covariates tested
☒	☐	A description of any assumptions or corrections, such as tests of normality and adjustment for multiple comparisons
☒	☐	A full description of the statistical parameters including central tendency (e.g. means) or other basic estimates (e.g. regression coefficient) AND variation (e.g. standard deviation) or associated estimates of uncertainty (e.g. confidence intervals)
☒	☐	For null hypothesis testing, the test statistic (e.g. F, t, r) with confidence intervals, effect sizes, degrees of freedom and P value noted<br>*Give P values as exact values whenever suitable.*
☒	☐	For Bayesian analysis, information on the choice of priors and Markov chain Monte Carlo settings
☒	☐	For hierarchical and complex designs, identification of the appropriate level for tests and full reporting of outcomes
☒	☐	Estimates of effect sizes (e.g. Cohen's d, Pearson's r), indicating how they were calculated

Our web collection on statistics for biologists contains articles on many of the points above.

Software and code

Policy information about availability of computer code

Data collection	Software used was HP Chemstation software (Rev. C.01.07 (27), Agilent Technologies) and Xcalibur software (version 3.0). No code was used.
Data analysis	The NIST mass spectral library (version 2.0) was used to analyse data. No code was used.

For manuscripts utilizing custom algorithms or software that are central to the research but not yet described in published literature, software must be made available to editors and reviewers. We strongly encourage code deposition in a community repository (e.g. GitHub). See the Nature Research guidelines for submitting code & software for further information.

Data

Policy information about availability of data

All manuscripts must include a data availability statement. This statement should provide the following information, where applicable:

- Accession codes, unique identifiers, or web links for publicly available datasets
- A list of figures that have associated raw data
- A description of any restrictions on data availability

The authors declare that all data supporting the findings of this study are available within the paper.

Field-specific reporting

Life sciences study design

All studies must disclose on these points even when the disclosure is negative.

Sample size	Selection of potsherd samples for analysis depended on how many sherds of a suitable size (c. 3-4g) for analysis were excavated from archaeological sites in the region. Of those that were suitable, a representative sample (at least 30 from each time period and vessel type) was chosen to reflect a range of time periods and vessel types. Thirty samples per time period provides a statistically reliable sample size.
Data exclusions	No data were excluded.
Replication	All samples were successfully run in duplicate.
Randomization	Not relevant - all samples were analysed.
Blinding	Blinding not relevant - this is not a controlled study. All samples underwent the same analysis.

Reporting for specific materials, systems and methods

We require information from authors about some types of materials, experimental systems and methods used in many studies. Here, indicate whether each material, system or method listed is relevant to your study. If you are not sure if a list item applies to your research, read the appropriate section before selecting a response.

Materials & experimental systems

n/a	Involved in the study	
☒	☐	Antibodies
☒	☐	Eukaryotic cell lines
☐	☒	Palaeontology and archaeology
☒	☐	Animals and other organisms
☒	☐	Human research participants
☒	☐	Clinical data
☒	☐	Dual use research of concern

Methods

n/a	Involved in the study	
☒	☐	ChIP-seq
☒	☐	Flow cytometry
☒	☐	MRI-based neuroimaging

Palaeontology and Archaeology

Specimen provenance	Pottery samples were obtained from collections at the University of Frankfurt. No permits were required.
Specimen deposition	Pottery samples will be returned to the University of Frankfurt. Samples are identified with the prefix NOK (for Nok culture) and are numbered 1-458
Dating methods	No new dates are provided.

☐ Tick this box to confirm that the raw and calibrated dates are available in the paper or in Supplementary Information.

Ethics oversight	No ethical approval is required to work on pottery samples.

Note that full information on the approval of the study protocol must also be provided in the manuscript.
